# Long-Term Follow-Up after Intravenous Immunoglobulin Therapy in Patients with Severe Ocular Mucous Membrane Pemphigoid Unresponsive to Conventional Therapy

**DOI:** 10.1155/2018/8372146

**Published:** 2018-09-19

**Authors:** Stefania Leuci, Massimo Amato, Elena Calabria, Raffaele Piscopo, Fausto Tranfa, Gianrico Spagnuolo, Michele Davide Mignogna

**Affiliations:** ^1^Oral Medicine Complex Unit, Department of Neuroscience, Reproductive and Odontostomatological Sciences, University “Federico II” of Naples, Naples, Italy; ^2^Dentistry Unit, Department of Medicine, Surgery and Dentistry, University of Salerno, Fisciano, Italy; ^3^Eye Center, Humanitas Research Hospital, Rozzano Milano, Italy; ^4^Orbital Unit, Department of Neuroscience, Reproductive and Odontostomatological Sciences, University “Federico II” of Naples, Italy; ^5^I. M. Sechenov First Moscow State Medical University, Institute of Dentistry, Moscow, Russia

## Abstract

Mucous membrane pemphigoid (MMP) is a heterogeneous group of rare, systemic, autoimmune subepidermal inflammatory disease that affects mucous membranes and the eye. In its most severe forms, this disease needs systemic therapy, usually based on steroids and immunosuppressant agents. In unresponsive cases or in the presence of contraindications or severe side effects due to conventional systemic corticosteroid and/or immunosuppressant therapy, a therapy shift to high-dose intravenous immunoglobulins (IVIg) has been recommended in other reports. This new therapy has proven to be effective in stopping ocular pemphigoid, but the data regarding the long-term effect on the disease activity or reactivation are extremely scarce, so the novel scientific aim of this study was to evaluate the clinical outcomes after a 9-year follow-up in 12 eyes (6 patients) affected by MMP with ocular involvement, successfully treated with IVIg therapy, as previously described in our report published in 2008. The evaluation of ocular and extraocular disease progression was performed at the end of IVIg therapy and at the end of the follow-up period. After 9 years, all the eyes enrolled showed a long-lasting remission of ocular and oral symptoms with a significant steroid-sparing effect. In conclusion, the IVIg has to be considered as a safe and successful alternative therapy in patients with severe ocular mucous membrane pemphigoid; furthermore, this kind of therapy seems to be effective in maintaining the clinical remission by the time.

## 1. Introduction

Mucous membrane pemphigoid (MMP) is a severe, systemic, autoimmune bullous disease that affects mucous membranes like ocular conjunctiva (64%), oral mucosa (85%), and occasionally the skin [1], which can have major morbidities and, rarely, deadly consequences [2–4].

Ocular MMP accounts for 61% of the cases of newly diagnosed cicatricial conjunctivitis between 60 and 80 years of age, with an incidence calculated as 0.8 per million population, and it affects women more often than men with a male-to-female ratio of nearly 2 : 1 [5]. Several studies have demonstrated an increased incidence of the HLA-DBQ1∗0301 allele in patients with MMP [6–8].

The main ocular sign of this autoimmune disease is a cicatricial symblepharon due to a subepithelial, complement-mediated inflammation caused by autoantibodies (IgG or IgA) directed to some antigen in the basement membrane [9].

Several studies demonstrated that the target antigens in the conjunctival basement membrane zone, such as antigen 180 (BP180) [10, 11], antigen 230 (BP230) [12], antigens 205 kd, 160 kd, 85 kd [13], laminin 5 (epilegrin) [14, 15], and *β*4-integrin [12, 16], and antigen 168 kd [17], are frequent in multiple mucosal sites and occasionally also in the skin.

The pathology produces a scar and it may affect the eye and other areas at the same time, in particular, the oral mucosa (85% of patients), the nasal mucosa (20–40%), the skin (25–30%), anogenital area and/or pharynx (20%), larynx (5–15%), and esophagus (5–15%) [5].

A subset of patients affected by MMP only suffer from ocular involvement: this peculiar MMP is known as ocular cicatricial pemphigoid (OCP) [9]. Both the MMP with ocular involvement and the OCP start with a conjunctival inflammation but in the latter stage the corneal scarring can lead to blindness [2].

Due to its severe scarring in the ocular, laryngeal, tracheal, oral, and esophageal involvement, the MMP may lead to a devastating course; hence, an aggressive therapy should be started immediately.

Systemic corticosteroids, together with the introduction of other immunosuppressive drugs, are the mainstay of treatment for severe MMP. Indications for systemic therapy include ocular disease unresponsive to less aggressive topical measures [4]. However, the high doses and prolonged administrations of corticosteroids that are often needed to control the disease can lead to many adverse, serious, and even life-threatening sequelae [4].

Alternative immunosuppressants such as cyclophosphamide, azathioprine, methotrexate, mycophenolate mofetil, dapsone, daclizumab, and mitomycin-C are also used [4, 18, 19], but some patients do not respond to these agents or they present serious adverse effects. In these unresponsive cases, the high dose of intravenous immunoglobulins (IVIg) therapy has been recommended thanks to its proven efficacy in several studies [20–25]; also our group showed a good result with this kind of therapy [26].

However, a challenge in the management of this kind of patients is to decide how much to prolong the IVIg therapy and also to assess the long-term effect on the ocular disease. In this study, on the basis of a previously published clinical trial on 6 patients successfully treated with IVIg [26], we report data about the long-lasting clinical remission during a nine-year follow-up since the last cycle of IVIg treatment.

## 2. Materials and Methods

An observational, retrospective, case-series study was conducted at the Oral Medicine Complex Unit, Department of Neuroscience, Reproductive and Odontostomatological Sciences, University Federico II of Naples, and was approved by the ethics committee. The study group consists of 12 eyes of 6 patients, who gave their informed consent to take part in the research study.

This cohort was the same included in our previous study published in 2008 [26]: three males and three females, who underwent to a nine-year follow-up. The age ranged from 58 to 80 (mean 69.5). All patients had initially at least ocular and oropharyngeal involvement. The diagnosis was made on the basis of clinical presentation, histology, immunopathological, and serological studies. All patients presented with bilateral conjunctival lesions, and all the eyes were classified into four stages ranging from I to IV according to Foster's classification [9]. For some patients, we also report the involvement of multiple mucosae and/or skin. One patient had nasal mucosal involvement, and two female patients and one male patient had genital lesions. Skin bullae were noticed by two patients. All clinical data are described in Table 1.

The inclusion criteria were based on the following:

  Clinically, the presence of active and/or progressive cicatricial inflammatory conjunctivitis (with the exclusion of any other possible pathology simulating the pemphigoid (pseudopemphigoid [27])), eventually associated with severe MMP manifesting as bullae/ulcers of the oropharyngeal
  Routine histology and direct immunofluorescence showing subepithelial split with mixed inflammatory cell infiltrate and deposits of IgG/C3 at dermoepidermal junction [2]; indirect immunofluorescence on salt split skin [28] and enzyme-linked immunosorbent assay (ELISA BP 180 and 230) positivity [29]
  The unresponsiveness to at least 6 months of conventional therapy and/or one or more contraindications to the use of high-dose long-term systemic corticosteroids (severe osteoporosis, diabetes, peptic ulcer disease, hypertension, and a previous myocardial infarction) [24]

The data examined in the study were collected and retrospectively reviewed in order to investigate the following:

  The previous treatments with relative side effects
  The IVIg protocol used
  The IVIg clinical response to and modality of therapy and adverse effects

In particular, with regard to previous therapies, we recorded the following data for each patient, before and during IVIg therapy: (1) the highest dose of corticosteroids, which was defined as the maximum dose per day a patient received to control MMP during the course of the disease; (2) the duration of corticosteroid therapy; (3) the number of relapses defined as appearance of 3 or more new lesions a month (skin blisters, oral mucosal erosions, and conjunctival lesions) which did not heal within 1 week, or the extension of a known conjunctival lesions in a patient who has achieved disease control [19]; and (4) the number of recurrences defined as reappearance of inflammation (which did not justify a change in systemic therapy) caused by the disease on a ocular or extraocular side already involved before the enrollment.

The relapse is well defined upon the criteria explained in the recommendations of an international panel of expert [19], while we also considered the recurrence in order to understand if the inflammation was totally controlled also in the OCP sites previously involved before the start of the therapy.

Side effects that were invariably expected from prolonged use of steroids and/or immunosuppressive agents were collected and classified into two groups: those which required medical treatments (glaucoma or cataract, osteoporosis, haemorrhagic cystitis, amenorrhoea, psychological reactions, steroid-induced acne, aseptic bone necrosis, and hyperglycaemia) and those which did not need any specific medical treatment (Cushing syndrome or insomnia).

Regarding the IVIg therapy, we studied the modality of IVIg therapy, the time to obtain clinical response, and the number of relapses or recurrence during or after the IVIg. As an evaluation of the clinical response, the patients had complete ophthalmological, mucosal, and skin examination at baseline and every 1-2 weeks.

From the ocular side, the effective clinical response to the therapy was considered as the absence of conjunctival inflammation (“white and quiet” eye) and the absence of progressive subepithelial fibrosis, while in the extraocular involvements, the clinical response was defined as effective if any healing and/or stopping of the progression of previous lesions was reported.

## 3. Results

### 3.1. Previous Therapies

The characteristics of the patients and the details of therapies (dose duration, side effects, etc.) are presented in Table 1.

In ocular lesions, topical steroids are ineffective in controlling the progression of disease [2]. Frequent lubrification and attention to eyelid hygiene are essential only to reduce symptoms and control secondary infections.

All the patients enrolled complained a progressive ocular conjunctival inflammation and an extensive oral involvement, so they required systemic therapy with corticosteroids (prednisone 1.5 mg/Kg/d) and one or more immunosuppressive agents (dapsone, azathioprine, and cyclophosphamide). Corticosteroid doses ranged from 60 mg to 100 mg/day with a cumulative total dose of steroids administered before IVIg therapy ranging from 16,200 mg to 81,000 mg. All patients received at least 1 adjuvant drug. Four patients developed side effects from conventional MMP therapy as described in Table 1. MMP conventional treatment and management of side effects required hospitalization in all patients. High-dose corticosteroids and immunosuppressive agents used in the management of patients before IVIg therapy were gradually tapered during the course of IVIg therapy. Corticosteroids and immunosuppressive agents used in the management of patients before IVIg therapy were given at highest dosage for at least 90 days and kept unchanged during the attack phase and then gradually tapered during the course of the treatment (maintenance phase).

### 3.2. IVIg Protocol and Patients Monitoring

The consensus statement of the use of intravenous immunoglobulin therapy in the treatment of autoimmune mucocutaneous blistering diseases describes how to assess the optimal dose, the length, and the frequency of the IVIg therapy [24].

The blood examinations at the baseline visit, prior to administer the IVIg cycle, were blood cell count, hepatic and renal function tests, plasma concentration of albumin and fibrinogen, lipid profile, routine urinalysis, serum levels of immunoglobulins specially IgA, rheumatoid factor, cryoglobulins, and antibodies to hepatitis B, C, and human immunodeficiency viruses. All the patients received, 30 minutes before the infusion, a medication with acetaminophen (500 mg), chlorpheniramine (20 mg), and methylprednisolone (40 mg) so as to limit a possible allergic reaction or side effect [24]. The cycle infusion consisted of IV human immunoglobulin, 5% solution (Flebogamma-Grifols, Ig vena NIV-Kedrion, Endobulin-Baxter) injected with an electronic pumping device (Optima MS, Fresenius Vial, France), and the dosage for each cycle 2 g was pro kg in three consecutive days [30, 31]. The treatment period before the IVIg ranged from 8 to 20 months, while the number of IVIg cycles varied from 10 to 20 (mean 16.8). The starting cycle frequency was every 2–4 weeks in the active phase.

Once the therapy showed a stop in the ocular MMP signs (like the stop of the conjunctival scarring and inflammation, or the healing of the extraocular MMP clinical signs), the interval time between each cycle was then gradually increased to 6, 8, 10, 12, 14, and 16 weeks (defined as maintenance therapy period).

In all 6 patients, the recurrence (range from 1 to 4) or the relapse (from 1 to 3) in this period gave us the indication to reduce the interval time between each cycle. Vital signs were monitored during the infusion. Before, during, and after each cycle, in order to assess all the therapy effects on MMP, every patient underwent to periodic follow-up visit in two or more of the following specialities (depending on the sites of the disease): dermatology, otolaryngology, odonthostomathology, gastroenterologist/endoscopist, clinical immunology, endocrinology, gynaecology, and nutritionist. In all patients, we carefully evaluate the coagulation profile and introduce preventive measures to reduce the risk of thromboembolic events such as acetyl-salicylic therapy (performed in all patients), as suggested by Katz and Shoenfeld, and subcutaneous calcium heparin (performed in 4 multimorbidity and multitherapy patients) [32, 33].

### 3.3. Clinical Response, Corticosteroid Tapering, and Adverse Effects of IVIg Therapy

All 12 eyes (6 patients) achieved effective clinical response in 5 to 12 months (mean 9.1) with IVIg therapy that was able to control active and progressive disease of the ocular and extraocular lesions and to arrest scarring (mean stage before IVIg therapy = 2.76 ± 0.49; mean stage after IVIg therapy = 2.42 ± 0.90). No extraocular new lesions or new areas of involvement were observed after the initiation of IVIg. These 6 patients have been maintained in a sustained remission for a total follow-up period of 9 years after the discontinuation of IVIg therapy. Successful clinical results previously described [26] have not changed, and no recurrence/relapse has been recorded throughout all the retrospective observational period.

## 4. Discussion

In this study, we report the successful long-term effects of IVIg therapy in the same 6 “high-risk” [4] MMP patients, earlier reported in our previous study [26], affected by ocular and extraocular involvement.

This kind of therapy has a well-established literature data supporting its effectiveness in ocular MMP [21–25], but studies about the long-term effect on the disease activity or reactivation are very scarce: in this study, the novel scientific content is to report the data about 9-year follow-up and, to the best of our knowledge, this is one of the longest follow-up time ever reported in the scientific literature.

All the patients enrolled were unresponsive or presented major contraindications to conventional therapies with high-dose oral corticosteroids in combination with immunosuppressants, so they were treated with IVIg.

Today, it is still unclear the mechanism by which the IVIg produces its effect in autoimmune disease, probably an initial anti-inflammatory effect is followed by the immunomodulation effect [24, 32, 34, 35]. The most recent studies [21–23, 29, 35–39] suggest the off-label IVIg use if the conventional therapy does not allow a good control of the disease or the contraindications of conventional therapy are remarkable. The use of IVIg has been proved to be effective in several chronic inflammatory or autoimmune diseases, such as Kawasaki's diseases, chronic inflammatory neuropathies, myasthenia gravis, dermatomyositis, and idiopathic thrombocytopenic purpura [36], and the prevalence of the life-threatening side effects is low if compared to systemic corticosteroids or immunosuppressant [37].

**Figure 1:**
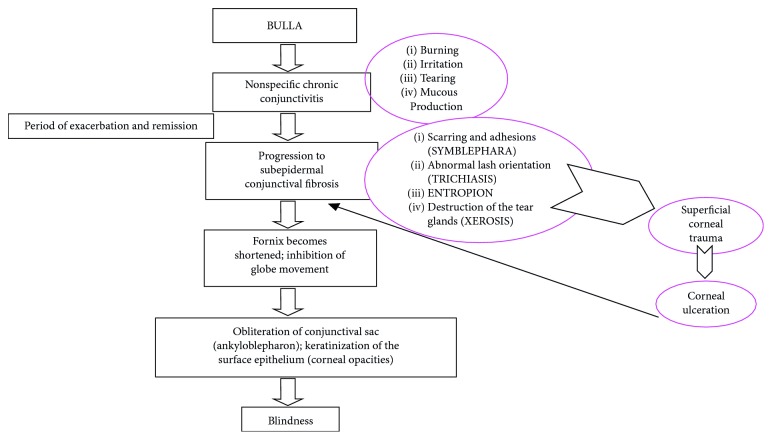
Synthetic clinical progression of the disease localized to conjunctiva.

In all 6 patients, the ocular signs were accompanied with gingival, buccal, and palate lesions as signs of MMP. One of the key features of the disease prognosis and follow-up is the early diagnosis and consequently early beginning of the therapy. When MMP appears as chronic conjunctivitis (“red eye”), specialists have difficulty in making a diagnosis in the early stages of the disease, and in many cases, MMP is not recognized until the disease process resulted in progressive scar formation and tissue contraction (symblepharon). The inferior fornix becomes shortened, and symblepharon formation increases to the point that the eyelids become firmly attached to the globe, inhibiting its movement. At later stages, the eyelids grow together and the conjunctival sac is obliterated (ankyloblepharon) (Figure 1). The progressive ocular disease can lead to blindness [1]. In oral cavity, the blisters quickly turn into ulcers that are frequent sites of secondary infection characterized by pain and result in poor nutrition. Healing reveals adhesions and scar formation. However, in exclusively oral involvement, the patients are defined as “low risk” in comparison with ocular, nasopharyngeal, esophageal, and laryngeal mucosa involvement [4, 5]. Therefore, the classification of patients in high and low risk is essential for clinician's decision on the correct modality and choice of treatment and drugs [5].

Among blistering disease, MMP is the most difficult to control: moreover, it may be a devastating systemic disease due to its severe ocular, laryngeal, tracheal, oral, and esophageal involvement.

The prognosis and the treatment of this pathology require early and very careful clinical and laboratory monitoring by a multidisciplinary team of specialists. IVIg appears to be safe [37–41], frequently associated with a variety of mild and temporarily adverse effects, such as low-grade fever, headache, nausea, myalgia, chills, arthralgias, flushing, abdominal cramps, and leucopenia. Although rare, the serious and potentially fatal side effects include anaphylactic reactions, aseptic meningitis, acute renal failure, stroke, myocardial infarction, and other thrombotic complications [42]. Many of these side effects have occurred in patients who have significant, underlying risk factors and comorbid conditions for the development of the event. A complete medical evaluation of the patient before the initiation of therapy is extremely important to identify risk factors that are associated with such side effects [37].

If compared to conventional therapy, also the IVIg needs a peculiar attention in several aspects (adequate patient selection, need of premedication, monitoring of patients, and modality of infusion) which are very important because they, if not well evaluated, can cause important and dangerous side effects.

As a consequence, the patient undergoing IVIg therapy needs a medical-multidisciplinary team which has to be used to handle this immunosuppressant drugs. Another limit of this therapy is the high cost: on this issue, we want to report a previous study from Daoud and Amin [43, 44] comparing the cost of the conventional therapy versus the IVIg: the authors point out that, even if the cost of the therapy is higher when the IVIg is used, the cost in relation on the side effects caused by both therapies is definitely lower in the IVIg therapy.

In our study, we reported a sustained long-term remission after discontinuation of IVIg for all of 6 patients, and no disease progression was observed. Similar outcome derives from Naveed Sami's study, in which 10 patients with progressive ocular-cicatricial pemphigoid (OCP), with severe refractory to conventional therapy and treated with IVIg therapy, have been maintained in a sustained remission for a total follow-up period ranging from 24 to 48 months (mean, 35) after discontinuing IVIg.

## 5. Conclusions

Overall, our data indicate that when current guidelines are followed, IVIg therapy could be able to rapidly control the activity of the disease producing a very effective clinical response together, allowing clinicians to taper consistently doses of corticosteroids and immunosuppressants. Further, IVIg therapy could be easily and safely performed in heavily pretreated MMP patients with only minor side effects and introduced as an alternative treatment modality in patients with severe disease in which long-lasting complete clinical remission could be achieved.

## Figures and Tables

**Table 1:** Demographic data and clinical details of patients pre- and post-IVIg.

Patient number	Age at onset/sex	Clinical profile	Duration of disease before IVIg (years)	Therapy before IVIg	Total steroids dose (mg)	Side effects of tx before IVIg	Number of relapses	Number of recurrences	Number of cycles of IVIg	Number of months of IVIg	Staging before IVIg	Staging after IVIg
1	64/F	Mouth, conjunctiva, vagina, skin	2	Prednisone 100 mg/day × 24 mo Azathioprine 150 mg/day × 24 mo Cyclophosphamide 100 mg/day × 20 mo	72,000 108,000 60,000	Osteoporosis, cataract, depression, anemia, leukopenia	0	3	20	18	RE: 3 LE: 3	RE: 3 LE: 3
2	58/F	Mouth, conjunctiva, nose, skin	3	Prednisone 90 mg/day × 36 mo Azathioprine 100 mg/day × 35 mo Cyclosporine (topical treatment) Methylprednisolone (topical treatment)	97,200 105,000	Anemia, leukopenia, Cushing syndrome	3	4	10	8	RE: 2 LE: 2	RE: 1 LE: 1
3	72/F	Mouth, conjunctiva, vagina	4	Prednisone 90 mg/day × 36 mo Cyclophosphamide 100 mg/day × 20 mo	97,200 60,000	Diabetes mellitus, hypertension	0	1	18	18	RE: 2 LE: 2	RE: 2 LE: 1
4	65/M	Mouth, conjunctiva	7	Prednisone 90 mg/day × 9 mo Azathioprine 100 mg/day × 7 mo Dapsone 75 mg/day × 60 mo	24,300 21,000 135,000	None	1	0	14	14	RE: 3 LE: 3	RE: 3 LE: 3
5	80/M	Mouth, conjunctiva	3	Prednisone 80 mg/day × 9 mo Azathioprine 150 mg/day × 6 mo Dapsone 75 mg/day × 12 mo	21,600 27,000 27,000	None	0	0	20	17	RE: 3 LE: 3	RE: 3 LE: 3
6	78/M	Mouth, conjunctiva, glans penis	10	Prednisone 80 mg/day × 20 mo Azathioprine 100 mg/day × 6 mo Cyclosporine (topical treatment)	48,000 18,000	Diabetes mellitus, leukopenia, hypertension	2	1	19	19	RE: 3 LE: 3	RE: 3 LE: 3

## Data Availability

All the data are available in Table 1 attached to the text file. Other data used to support the findings of this study are available from the corresponding author upon request.
